# Bariatric surgery blunts nitrate-mediated improvements in cardiovascular function of overweight women by interfering with gastric S-nitrosothiol formation

**DOI:** 10.1016/j.redox.2024.103440

**Published:** 2024-11-24

**Authors:** Jéssica Maria Sanches-Lopes, Alessandra Cássia-Barros, Sandra Oliveira Conde-Tella, Eduardo Barbosa Coelho, Rafael Kemp, Riccardo Lacchini, Martin Feelisch, Wilson Salgado Júnior, Jose Eduardo Tanus-Santos

**Affiliations:** a Department of Pharmacology, Ribeirao Preto Medical School, University of Sao Paulo, Ribeirao Preto, SP, Brazil; b Department of Translational Medicine, State University of Campinas, Campinas, SP, Brazil; c Department of Medicine, Ribeirao Preto Medical School, University of Sao Paulo, Ribeirao Preto, SP, Brazil; d Department of Surgery and Anatomy, Ribeirao Preto Medical School, University of Sao Paulo, Ribeirao Preto, SP, Brazil; e Department of Psychiatric Nursing and Human Sciences, Ribeirao Preto College of Nursing, University of Sao Paulo, Brazil; f Clinical & Experimental Sciences, Faculty of Medicine, University of Southampton, UK; g Southampton NIHR Biomedical Research Centre, University Hospital Southampton NHS Foundation Trust, UK

**Keywords:** Cardiovascular, Nitrite, Nitrate, Nitrosation, Endothelial function, S-nitrosothiols

## Abstract

Inorganic nitrate (NO_3_^−^) and nitrate-rich foods have been shown to exert antioxidative effects and lower blood pressure in experimental animal models and human clinical studies. The specific handling of nitrate, including its enterosalivary recirculation, secretion into saliva, oral microbial reduction to nitrite (NO_2_^−^), and the pH-dependent nitrosative capacity in the stomach have all been recognized as being important for nitrate's beneficial effects. Obesity is of major health concern worldwide and associated with increased cardiovascular risk; whether nitrate lowers blood pressure and improves endothelial function in this setting has not been investigated. We here tested the hypotheses that i) nitrate elicits cardiovascular benefits in overweight women; and ii) these beneficial effects would be diminished in women who underwent bariatric Roux-en-Y gastric bypass (RYGB) surgery. Our controlled clinical trial included 15 women with prior RYGB surgery and 15 overweight female controls. All participants received a single dose of 0.1 mmol/kg/day nitrate in the form of a beetroot extract for 14 days. Blood collection, 24-h ambulatory blood pressure measurements and endothelial function tests were performed before and after nitrate treatment. Plasma nitrite, nitrate, and S-nitrosothiol (RSNO) concentrations were determined by ozone-based reductive chemiluminescence while thiobarbituric acid reactive substances (TBARS) and total antioxidant capacity (TAC) were measured using plate-reader based assays. Nitrate reduced blood pressure and improved endothelial function in controls, but not in women with prior bariatric surgery. Nitrate also increased circulating nitrate/nitrite and RSNO levels in controls, but the latter was blunted following RYGB surgery despite even larger increases in nitrite concentrations. Similarly, nitrate increased antioxidant responses in controls but not in women with prior bariatric surgery. This is the first study to show that nitrate exerts beneficial cardiovascular effects in obesity and that the morphological/functional modifications elicited by RYGB surgery abrogates nitrate's effectiveness by preventing gastric RSNO formation.

## Highlights

• Nitrate-rich foods can reduce blood pressure and have antioxidative effects.
• We examined nitrate's benefits in overweight women and post-bariatric surgery.
• Nitrate improved blood pressure and endothelial function only in controls.
• Bariatric surgery prevented S-nitrosothiols formation after nitrate therapy.
• Bariatric surgery lessens nitrate's cardiovascular effects due to gastric changes.

## Graphical abstract

**Figure:**
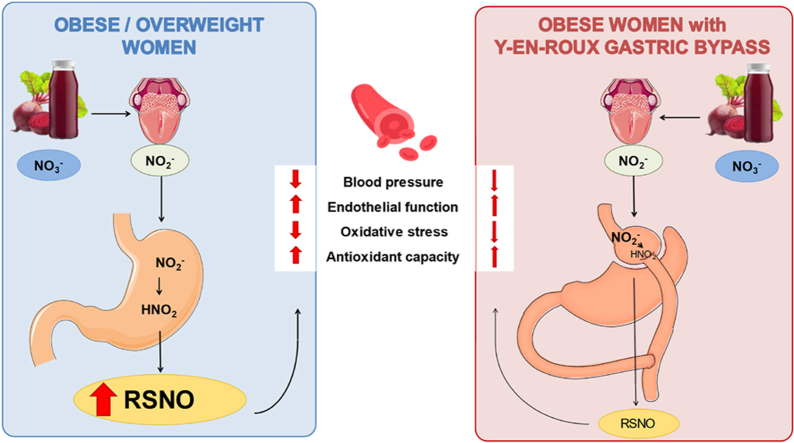

## 1 Introduction

The free radical nitric oxide (NO) has long been acknowledged as a critical player in maintaining the function of the cardiovascular system and numerous other physiological processes [1]. Indeed, NO deficiency usually causes diseases as it severely impairs vascular function and blood pressure regulation, platelet aggregation and leukocyte adhesion among other important roles affecting metabolic regulation, neurotransmission, and immunity [2]. Based on this idea, strategies to increase NO bioavailability have been proposed to treat various disease conditions, and because NO is endogenously produced from l-arginine by NO synthases, drugs that upregulate these enzymes have shown beneficial effects [3,4]. Alternatively, NO can also be generated via the nitrate-nitrite-NO pathway as a result of dietary supplementation with nitrate (NO_3_^−^) or nitrite (NO_2_^−^) causing therapeutic effects, particularly in cardiovascular and metabolic diseases [[5], [6], [7], [8]], an approach exploited for centuries without knowledge of the exact mode of action of these simple inorganic compounds [9].

Dietary nitrate requires bioactivation to nitrite and then to NO in order to produce its beneficial effects. This is usually accomplished by entering the enterosalivary recirculation of endogenously produced nitrate and has been regarded as a key mechanism to stimulate NO formation in many disease conditions [10]. In this cycle, nitrate is taken up by the salivary glands and secreted into saliva, where it is reduced to nitrite by oral commensal bacteria possessing nitrate-reductase activity [11]. Swallowed nitrite, in turn, was shown in seminal studies published more than three decades ago to generate NO in a non-enzymatic manner in the acidic environment of the stomach [12,13]. Importantly, it is now clear that the chemical process of disproportionation following the protonation of nitrite to nitrous acid in the stomach gives rise to the gastric formation of a variety of reactive nitrogen oxides (including HNO_2_, N_2_O_3,_ and NO_2_) that act as [NO^+^] carriers and may react with deprotonated thiols (RS^−^) to generate S-nitrosothiols (RSNO) [[14], [15], [16]]. This mechanism of endogenous RSNO formation from nitrite critically depends on low gastric pH, and recent studies using proton pump inhibitors or other inhibitors of gastric acid secretion, which increase gastric pH, demonstrated that these drugs prevent both the formation of RSNO and the hypotensive effects of orally administered nitrite [16,17]. However, it cannot be excluded that proton pump inhibitors may affect RSNO levels via effects other than raising gastric pH. Moreover, recent clinical studies have consistently shown that treatment with nitrate, which is bioactivated to nitrite in the course of the enterosalivary circulation of nitrate [10], increases circulating RSNO concentrations [18] and this response directly correlates with cardiovascular effects [19,20], thus suggesting a major role for RSNO formation in the cardiovascular responses to nitrate administration [21]. Therefore, any factor that possibly impairs RSNO formation after oral nitrate or nitrite administration is likely to prevent the beneficial cardiovascular effects associated with this approach [22]. In fact, growing evidence suggests that impairing RSNO formation may prevent the long-lasting functional effects of RSNO-mediated nitrosation [23], an important post-translational protein modification that regulates cardiovascular function [24,25] and thus a relevant pharmacological target [26,27].

Because overweight and obesity pose a major public health problem worldwide, many therapeutic options have been proposed including lifestyle modifications, pharmacotherapy, and bariatric surgery [28]. The latter option may be chosen depending on the severity of the disease and functional limitations and offers major advantages such as weight loss and improvements in obesity-related conditions including reduction in cardiovascular risk [28]. However, a subset of patients may also face some harm, particularly with the so-called Roux-en-Y gastric bypass (RYGB) surgery, which reduces food intake by creating a small pouch in the upper part of the stomach, which is connected to a Roux limb of the jejunum [28]. While RYGB surgery allows food to bypass most of the stomach, the duodenum, and part of the jejunum, an unintended consequence of this intervention in that gastric pH increases significantly. Indeed, it has been shown that the pre-surgery gastric pH of 1.8 increases to 6.4 after RYGB surgery, with major implications for gastric pH-dependent drug dissolution [29] and other pharmacological responses [30,31]. Whether RYGB surgery (with increased gastric pH) affects the disproportionation of nitrite in the stomach and RSNO levels in the circulation is unknown.

The present study tested the hypotheses that i) oral nitrate administration in the form of a beetroot extract exerts favorable cardiovascular effects on endothelial function and blood pressure by increasing systemic RSNO concentrations and ii) that the morphological and functional modifications of the gastrointestinal system associated with RYGB surgery have a major impact on the bioactivation of nitrate and the associated cardiovascular responses by impairing RSNO formation.

## 2 Materials and methods

### 2.1 Ethical aspects

This study was approved by the Institutional Review Board (CAAE number: 88796918.2.000.5440) of the Hospital das Clinicas of Ribeirao Preto Medical School, University of Sao Paulo, Brazil, and each participant signed a written free and informed consent form. The study was carried out in accordance with the ethical standards of the Helsinki Declaration and registered in ClinicalTrials.gov (NCT06303830).

### 2.2 Study participants

This study is a controlled clinical trial (interventional study) that included overweight women who either underwent RYGB surgery (bariatric surgery group, N = 15) or not (controls, without prior surgery; N = 15). Both groups received the same treatment, allowing for a direct comparison of outcomes between the two cohorts. The inclusion criteria were: women, 18–60 years, with stable body weight for at least 6 months, and those in the bariatric surgery group must have undergone the RYGB surgery at least 1.5 years prior to enrolment in the study. The RYGB technique standardized in the service was performed with a small 20 mL gastric pouch, a gastrojejunal anastomosis 1.5–2 cm in diameter and with biliopancreatic and alimentary loops of 100 cm each. The exclusion criteria for both groups included hypertensive patients taking more than two anti-hypertensive agents, uncontrolled hypertension (blood pressure >160/100 mmHg, even if regularly taking two anti-hypertensive drugs), diabetes mellitus or any other endocrinopathy, renal failure, liver diseases, and smoking. The sample size estimate was based on a previous study that evaluated the effect of sodium nitrite treatment on plasma RSNO concentrations in patients [32]. A statistical power of 80 % and an alpha of 5 % were used, along with the difference between the mean plasma concentration of RSNOs before (15 ± 11 nM) and after (26 ± 18 nM) sodium nitrite administration observed in this previous study [32]. The calculation was performed using G∗Power 3.1.9.2 software and indicated an estimated sample size of 15 participants per group.

### 2.3 Study design

On the first day of the study, participants visited the Clinical Research Unit (CRU) in the Hospital das Clínicas of Ribeirao Preto Medical School/USP after overnight fasting. Endothelial function was evaluated as described below, followed by collection of venous blood to analyze cholesterol, triglycerides, HDL, LDL, uric acid, blood glucose and creatinine concentrations. Additional blood samples were collected into heparin containing vacutainer tubes (Becton-Dickinson Sao Paulo, Brazil), plasma was obtained by centrifugation at 1000×*g* for 10 min, and aliquoted into tubes containing 10 mM N-ethylmaleimide (NEM) and 2 mM diethylenetriamine pentaacetic acid (DTPA) for the preservation of RSNO content [33]. Thereafter plasma samples were immediately stored at −70 °C until biochemical analysis.

In order to assess baseline blood pressure before start of the nitrate treatment an ambulatory blood pressure monitoring (ABPM) device (DynaMapa, Cardios, São Paulo, Brazil) with proper arm cuff was fitted to each participant to assess blood pressure over a 24-h period, with measurements taken every 15 min during daytime and every 30 min during nighttime. The following day, participants returned to the hospital for removal of the ABPM device and data retrieval. Then each participant started their daily treatment with a nitrate-enriched beetroot extract dissolved in about 200 mL of water once every morning, for 14 days. Each dose was individually prepared and comprised 5 g of the original beetroot extract (Florien Fitoativos, Piracicaba, SP, Brazil), which contained 1.5 g of nitrate per 100 g, to which sodium nitrate was added in amounts to allow exactly 0.1 mmol/kg of nitrate to be administered to each study participant per day. Participants were instructed not to modify their dietary habits and not to use any drug or substance that could affect the enterosalivary cycle of nitrate (such as inhibitors of gastric acid secretion or antiseptic mouthwash).

After 14 days of nitrate treatment, the participants returned to the CRU after an overnight fast. Endothelial function was assessed again, and then the ABPM device was attached again, the participant received their last dose of nitrate-enriched beetroot extract, and blood samples were collected for the last time exactly 2 h and 30 min after nitrate intake. The following day, the participants returned to the CRU and the ABPM device was removed which concluded the study.

### 2.4 Assessment of endothelial function by finger pulse plethysmography

The EndoPAT 2000 device (Itamar Medical Ltd, Caesarea, Israel) is a non-invasive diagnostic device used to assess endothelial function by measuring the reactive hyperemia index (RHI) following a brief occlusion of blood flow, which reflects endothelial health. The test was carried out after a 20 min rest period and is based on the principle of peripheral arterial tonometry (to assess pulsatile arterial volume changes), using finger-mounted pneumatic probes to detect changes in arterial pulse volume. Baseline measurement are carried out with the patient wearing fingertip probes on both hands. These probes measure the baseline pulse wave amplitude (PWA), which reflects normal blood flow in the arteries. Then a blood pressure cuff is placed on one arm and inflated to occlude blood flow in the brachial artery for 300 s. This period of occlusion creates a condition of ischemia in the arm. After releasing the cuff, blood flow returns rapidly, causing shear stress-induced reactive hyperemia. The EndoPAT device measures the changes in pulse wave amplitude during this period, reflecting how well the endothelium responds to the increased blood flow. The results are reported as the natural logarithm of RHI, with lower values indicating endothelial dysfunction. The dominant arm serves as an internal control to correct for any systemic drift in vascular tone during the test [34].

### 2.5 Measurement of plasma nitrite, nitroso compounds (RXNO), S-nitrosothiols (RSNO), and nitrate concentrations

Plasma aliquots were analyzed in duplicate for their nitrite and nitrosated (RXNO and RSNO) species contents using an ozone-based reductive chemiluminescence assay as previously described [35,36]. Briefly, to measure nitrite concentrations in plasma, 100 μl of plasma samples were injected into a solution of acidified tri-iodide, purged with nitrogen in line with a gas-phase chemiluminescence NO analyzer (Sievers Model 280 NO analyzer; Boulder, CO, USA).

To measure plasma nitroso compounds (RXNO) concentrations, 450 μl of plasma samples were treated with acidic sulfanilamide (5 % sulfanilamide in HCl 1 mol/L) for 3 min before injection into the solution of acidified tri-iodide purged with nitrogen in line with the NO analyzer. To discriminate RSNO from other NO-related species, subtractive measurements were carried out using samples treated with mercuric chloride (HgCl_2_, 2 %) for 2 min followed by acidic sulfanilamide for 3 min before injection into acidified tri-iodide solution [35,36].

To measure plasma nitrate concentrations, 20 μl of plasma samples were analyzed in duplicate for their nitrate levels by injection into a solution of vanadium (III) chloride in 1 M hydrochloric acid at 95 °C [36], purged with nitrogen in line with the same gas-phase chemiluminescence NO analyzer as above. Areas under the curves (AUCs) of the NO signals obtained from each analysis configuration were integrated and compared to the AUC of freshly prepared aqueous nitrite standards.

### 2.6 Assessment of plasma lipid peroxide concentrations and total antioxidant capacity

Previous studies have shown that nitrate therapy results in antioxidant responses [[37], [38], [39], [40]]. Therefore, we studied select markers of oxidative stress in the present study to examine whether the RYGB bariatric surgery would affect these readouts in response to nitrate. Plasma lipid peroxide levels were determined by measuring thiobarbituric acid reactive substances (TBARS) using a previously detailed method [41]. Lipid peroxide levels were expressed as malondialdehyde (MDA) equivalents (nmol/mL). Briefly, plasma samples were incubated for 1 h at 95 °C in the presence of acetic acid (pH 3.5), sodium dodecyl sulfate 10 % and thiobarbituric acid 0.6 %. After this, the incubates were centrifuged for 10 min at 12,000×*g*, and the supernatants were collected and read spectrophotometrically at 532 nm using malondialdehyde as standard [41].

Total Antioxidant Capacity (TAC) was measured in plasma samples using a commercially available assay kit (#709001, Cayman Chemical, Ann Arbor, MI, USA), which is based on the ability of plasma antioxidants to inhibit the oxidation of ABTS (2,2′-azino-di-[3-ethylbenzthiazoline sulfonate]) [42]. In this method, ABTS reacts with hydrogen peroxide (H₂O₂) in the presence of metmyoglobin to produce a radical cation, which has a distinct color. Antioxidants suppress this color development in proportion to their concentration in plasma. The absorbance was measured at 750 nm (μQuant microplate reader; Bio-Tek Instruments Inc., Winooski, VT, USA), and results were compared to a Trolox (a water-soluble analog of vitamin E; 6-hydroxy-2,5,7,8-tetramethylchroman-2-carboxylic acid) standard curve to quantify the antioxidant capacity.

### 2.7 Statistical analysis

The results are expressed as mean ± S.D. and were analyzed by using unpaired *t*-test or two-way ANOVA for repeated measures, with the two factors being treatment and bariatric surgery, followed by Bonferroni post-hoc test. Pearson's correlation test was also performed. The statistical significance level was set at 95 % (p < 0.05). All statistical analysis were performed using GraphPad Prism 8 software.

## 3 Results

### 3.1 Clinical and laboratory characteristics of study groups

Our study cohort consisted of 30 overweight women, 15 women in the control group and 15 women who underwent RYGB surgery prior to enrolment. Both groups showed similar anthropometric and laboratory characteristics, including age, height, weight, body mass index (BMI), cholesterol, triglycerides, HDL, LDL, uric acid, blood glucose and creatinine (Table 1; all P > 0.05), indicating that they were comparable. Although subjects in the bariatric surgery group had a mean BMI >30 and were obese, their average BMI was not statistically different from that of the control group (Table 1; P > 0.05). There were two hypertensive women in the control group, and one in the bariatric surgery group (P > 0.05).

**Table 1:** Clinical and laboratory characteristics of women in both RYGB surgery (Bariatric Surgery group, N = 15) and overweight women (Control group, without prior surgery; N = 15).

	Control	Bariatric Surgery	P Value
**Age (years)**	41.9 ± 9.8	47.3 ± 11.5	0.173
**Weight (Kg)**	75.5 ± 15.9	78.6 ± 12.4	0.552
**Height (m)**	1.63 ± 0.05	1.59 ± 0.08	0.152
**BMI (Kg/m**^**2**^**)**	28.2 ± 5.9	31.0 ± 4.2	0.140
**Cholesterol (mg/dL)**	189 ± 21	183 ± 28	0.589
**Triglycerides (mg/dL)**	86 ± 25	89 ± 34	0.077
**HDL (mg/dL)**	55 ± 8	59 ± 13	0.374
**LDL (mg/dL)**	119 ± 23	106 ± 23	0.208
**Uric Acid (mg/dL)**	3.4 ± 0.8	4.0 ± 1.1	0.114
**Blood Glucose (mg/dL)**	84 ± 5	85 ± 9	0.737
**Creatinine (mg/dl)**	0.72 ± 0.14	0.67 ± 0.10	0.296

The results are expressed as mean ± S.D.

BMI, body mass index.

### 3.2 Nitrate treatment reduces blood pressure in overweight women, but not in those with prior RYGB bariatric surgery

**Fig. 1:**
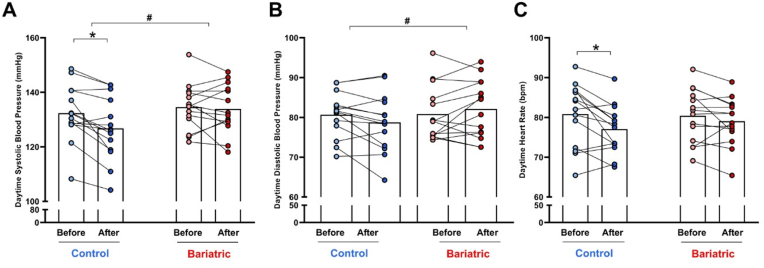
**RYGB bariatric surgery prevents the blood pressure lowering effects of nitrate therapy.** Daytime systolic blood pressure (panel A), diastolic blood pressure (panel B), and heart rate (panel C) are shown in the control and bariatric surgery groups before (light colors) and after (dark colors) nitrate treatment for 14 days. Data are shown as mean (N = 15 per group). ∗P < 0.05 for After *vs.* Before nitrate treatment. # Significant interaction between factors (treatment and bariatric surgery) as indicated by the 2-way ANOVA.

In order to obtain the best information regarding blood pressure effects, we used 24-h ABPM. Nitrate treatment for 14 days decreased daytime systolic blood pressure in the control group (baseline = 132 ± 10 mmHg *vs.* 126 ± 11 mmHg after nitrate; P < 0.05; Fig. 1A), but not in the bariatric surgery group (baseline = 135 ± 8 mmHg *vs.* 134 ± 9 mmHg, P > 0.05; Fig. 1A). However, we found no significant effects of nitrate treatment on daytime diastolic blood pressure in either the control group (baseline = 81 ± 6 mmHg *vs.* 79 ± 7 mmHg after nitrate; P > 0.05; Fig. 1B) or the bariatric surgery group (baseline = 81 ± 7 mmHg *vs.* 82 ± 7 mmHg, P > 0.05; Fig. 1B), although 2-way ANOVA showed a significant interaction between treatment and bariatric surgery (P < 0.05; Fig. 1B). Similar effects were seen when we analyzed mean arterial pressure (not shown). Nitrate did not have any significant effects on night-time blood pressure (data not shown; all P > 0.05). Nitrate treatment also reduced daytime heart rate in the control group (baseline = 81 ± 8 mmHg *vs.* 77 ± 6 mmHg after nitrate; P < 0.05; Fig. 1C), but not in the bariatric surgery group (baseline = 80 ± 7 mmHg *vs.* 79 ± 6 mmHg, P > 0.05; Fig. 1C).

### 3.3 Nitrate treatment improves endothelial function in overweight women, but not in those with prior RYGB bariatric surgery

**Fig. 2:**
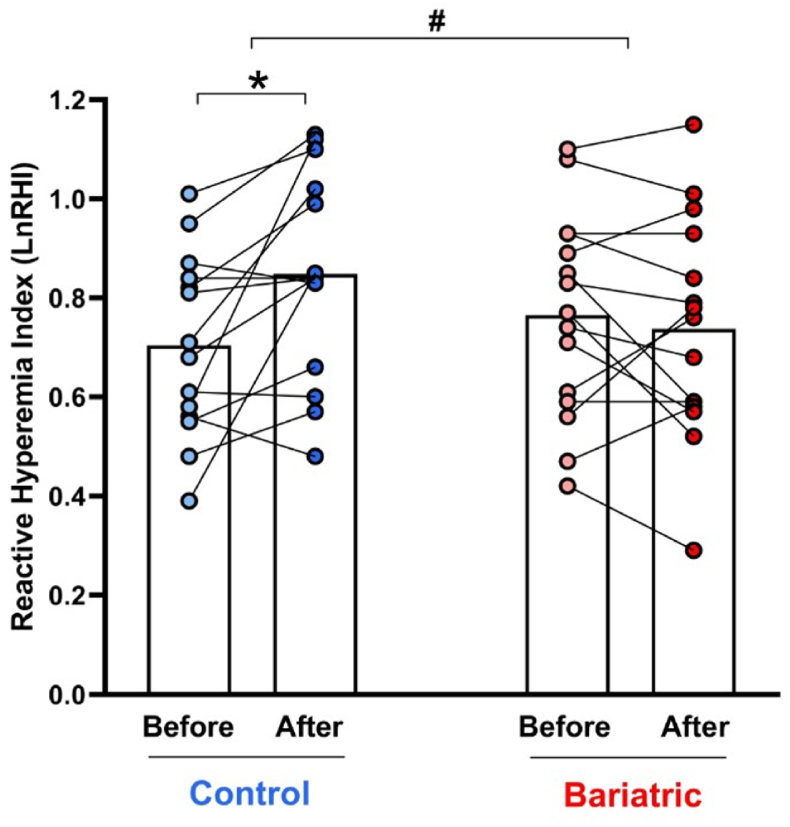
**RYGB bariatric surgery prevents nitrate therapy-induced improvement in endothelial function.** Endothelial function was assessed by finger plethysmography with EndoPAT in the control and bariatric surgery groups before (light colors) and after (dark colors) nitrate treatment for 14 days. Data are shown as mean (N = 15 per group). ∗P < 0.05 for After *vs.* Before nitrate treatment. # Significant interaction between factors (treatment and bariatric surgery) as indicated by the 2-way ANOVA.

The assessment of endothelial function by finger plethysmography using EndoPAT showed that nitrate treatment improved the reactive hyperemia index in the control group (baseline = 0.70 ± 0.18 *vs.* 0.85 ± 0.21 after nitrate; P < 0.05; Fig. 2), but not in the bariatric surgery group (baseline = 0.77 ± 0.21 *vs.* 0.74 ± 0.22 after nitrate; P < 0.05; Fig. 2), with a significant interaction between treatment and bariatric surgery (P < 0.05; Fig. 2).

### 3.4 While nitrate treatment increased NO metabolite concentrations in overweight controls, prior RYGB bariatric surgery prevented the increases in RXNO and RSNO concentrations

**Fig. 3:**
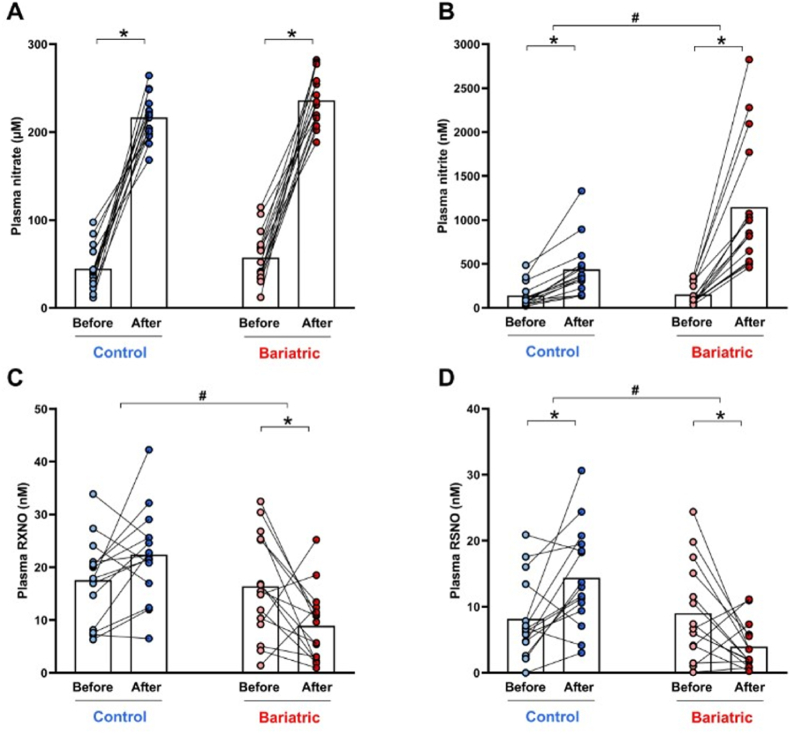
**RYGB bariatric surgery prevents nitrate therapy-induced increases in plasma nitroso compounds (RXNO), and S-nitrosothiols (RSNO).** Plasma nitrate (Panel A), nitrite (Panel B), RXNO, and RSNO were measured by ozone-based chemiluminescence assays in samples from the participants in the control and bariatric surgery groups before (light colors) and after nitrate (dark colors) treatment for 14 days. Data are shown as mean (N = 15 per group). ∗P < 0.05 for After *vs.* Before nitrate treatment. # Significant interaction between factors (treatment and bariatric surgery) as indicated by the 2-way ANOVA.

Nitrate treatment resulted in similar increases in plasma nitrate concentrations in controls (baseline = 45 ± 25 μM *vs.* 216 ± 26 μM after nitrate; P < 0.05; Fig. 3A) and in the bariatric surgery group (baseline = 57 ± 29 μM *vs.* 236 ± 29 μM after nitrate; P < 0.05; Fig. 3A).

Plasma nitrite concentrations also increased in both study groups (baseline = 140 ± 136 nM *vs.* 438 ± 315 nM after nitrate in the control group; and baseline = 152 ± 120 nM *vs.* 1147 ± 739 nM after nitrate in the bariatric group; both P < 0.05; Fig. 3B), with considerably higher increases in the bariatric compared to the control group (7.5-fold increase versus 3.1 increase; P < 0.05; Fig. 3B). In contrast to these changes in plasma nitrite and nitrate concentrations, plasma RXNO levels changed into opposite directions, tending to increase in the control group (baseline = 17.6 ± 7.9 nM *vs.* 22.4 ± 8.7 nM after nitrate; P > 0.05; Fig. 3C) while clearly decreasing in the bariatric surgery group (baseline = 16.4 ± 9.8 nM *vs.* 8.9 ± 6.9 nM after nitrate; P < 0.05; Fig. 3C). Similarly, we found an interaction between treatment and bariatric surgery on RSNO concentrations, which increased in the control group (baseline = 8.2 ± 6.0 nM *vs.* 14.4 ± 7.6 nM after nitrate; P < 0.05; Fig. 3D), and decreased in the bariatric surgery group (baseline = 9.0 ± 7.3 nM *vs.* 4.0 ± 3.5 nM after nitrate; P < 0.05; Fig. 3D). Changes in mercury stable NO-adducts concentrations, obtained by subtraction of RSNO from RXNO levels, did not show significant differences (P > 0.05; data not shown).

### 3.5 Nitrate treatment lowered lipid oxidation and increased antioxidant capacity in overweight controls, and prior RYGB bariatric surgery prevented these effects

**Fig. 4:**
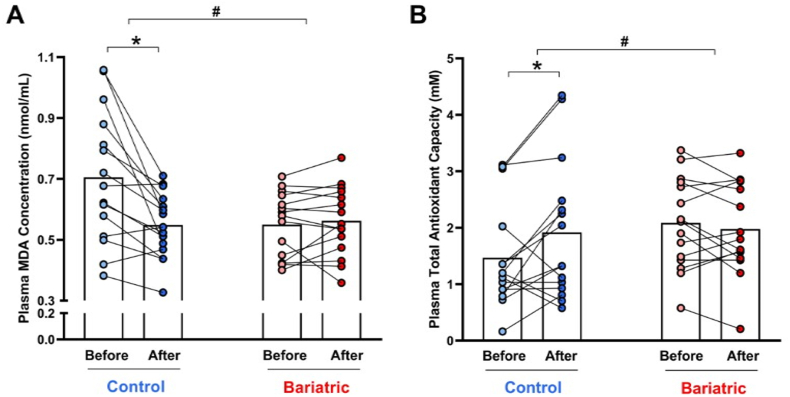
**RYGB bariatric surgery prevents nitrate therapy-induced antioxidant effects.** Plasma lipid peroxide levels were determined by measuring thiobarbituric acid reactive substances and expressed in terms of malondialdehyde (MDA; Panel A) (nmol/mL). The plasma Total Antioxidant Capacity (TAC; Panel B) was measured in plasma samples using a commercially available kit. Both measurements were carried out in samples from the participants in the control and bariatric surgery groups before (light colors) and after (dark colors) nitrate treatment for 14 days. Data are shown as mean (N = 15 per group). ∗P < 0.05 for After *vs.* Before nitrate treatment. # Significant interaction between factors (treatment and bariatric surgery) as indicated by the 2-way ANOVA.

TBARS concentrations and TAC were measured in plasma samples to examine the effects of nitrate treatment on oxidative stress-related readouts. We found an interaction between treatment and bariatric surgery on TBARS concentrations, which decreased in the control group (baseline = 0.71 ± 0.22 nmol/mL *vs.* 0.55 ± 0.11 nmol/mL after nitrate; P < 0.05; Fig. 4A), but did not change in the bariatric surgery group (baseline = 0.55 ± 0.11 nmol/mL *vs.* 0.56 ± 0.11 nmol/mL after nitrate; P > 0.05; Fig. 4A). These results demonstrate that nitrate therapy decreased oxidative stress in controls but not in participants with prior RYGB bariatric surgery. In agreement with these results, we found that TAC increased in the control group (baseline = 1.47 ± 0.92 mM *vs.* 1.92 ± 1.24 mM after nitrate; P < 0.05; Fig. 4B), and did not change in the bariatric surgery group (baseline = 2.08 ± 0.81 mM *vs.* 1.98 ± 0.82 mM after nitrate; P > 0.05; Fig. 4B). Together, these results indicate that nitrate therapy resulted in antioxidant responses in overweight women but not in participants with prior RYGB bariatric surgery.

### 3.6 Endothelial function (LnRHI) is directly associated with plasma S-nitrosothiols (RSNO) concentrations, whereas daytime systolic blood pressure is inversely associated with RSNO concentrations

**Fig. 5:**
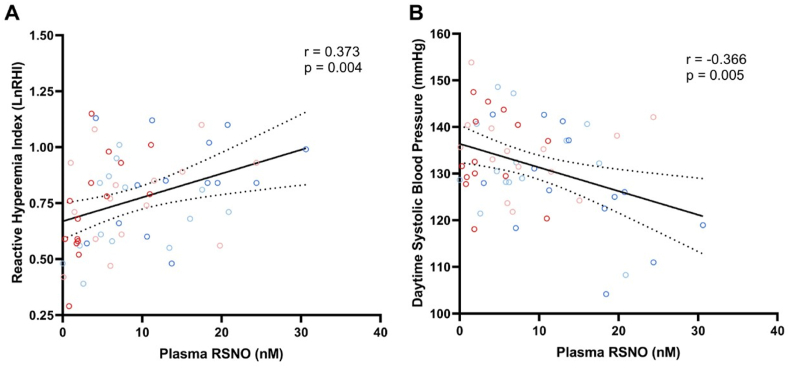
**The endothelial function (LnRHI) is directly associated with plasma S-nitrosothiols (RSNO) concentrations, whereas daytime systolic blood pressure is inversely associated with RSNO concentrations.** Reactive Hyperemia Index (LnRHI; panel A), daytime systolic blood pressure (panelB), and plasma RSNO concentrations measured in controls (blue) and in participants with prior RYGB bariatric surgery (red) before (light colors) and after (dark colors) nitrate therapy are shown. r = Pearson's correlation coefficient. The regression line and the 95 % confidence interval were plotted.

The Reactive Hyperemia Index (LnRHI) reflects the endothelial function and we found a significant (albeit not strong) positive correlation between LnRHI and RSNO concentrations (Fig. 5A; r = 0.373; P = 0.004), and an inverse correlation between daytime systolic blood pressure and RSNO (Fig. 5B; r = −0.366; P = 0.005), suggestive of a significant association between increases in RSNO and improved endothelial function as well as decreased systolic blood pressure.

### 3.7 Plasma total antioxidant capacity (TAC) is directly associated with plasma nitrite concentrations, and moderately associated with RNSO concentrations

**Fig. 6:**
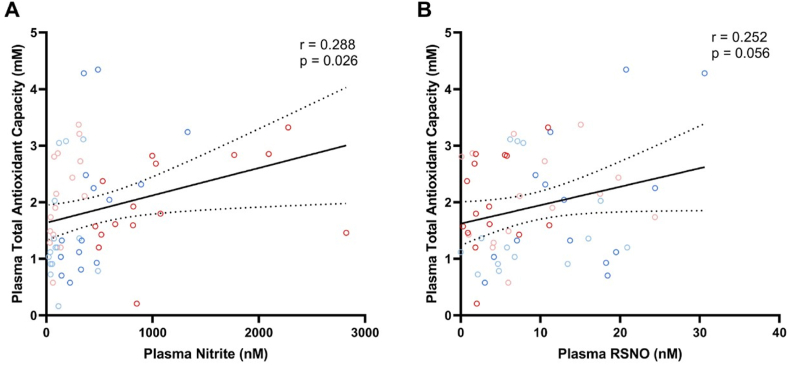
**The plasma total antioxidant capacity (TAC) is directly associated with plasma nitrite concentrations, and tended to be directly associated with RNSO concentrations.** Plasma TAC, nitrite (panel A), and RSNO (panel B) concentrations measured in controls (blue) and in participants with prior RYGB bariatric surgery (red) before (light colors) and after (dark colors) nitrate therapy are shown. r = Pearson's correlation coefficient. The regression line and the 95 % confidence interval were plotted.

Plasma TAC was positively correlated with plasma nitrite concentrations (Fig. 6A; r = 0.288; P = 0.026), and tended to correlate positively with plasma RSNO concentrations (Fig. 6B; r = 0.252; P = 0.056), thus suggesting a significant association between increases in plasma nitrite (and RSNO) and antioxidant responses.

## 4 Discussion

This is the first study to demonstrate that i) a 14-day treatment with dietary nitrate improves endothelial function and lowers systolic blood pressure in overweight women, and that ii) the morphological and functional modifications induced by RYGB bariatric surgery result in major changes to the metabolic bioactivation of nitrite, severely impair RSNO formation, and blunt the beneficial cardiovascular effects of a treatment with nitrate-enriched beetroot extract. These changes are likely attributable to the significant alterations in gastric pH caused by RYGB surgery [29] and known to affect drug responses [30,31].

Treatment with nitrate resulted in a statistically significant and clinically meaningful decrease in daytime systolic blood pressure (about 6 mmHg) and heart rate (about 4 bpm) in the control group. Importantly, to assess blood pressure we used 24-h ABPM, which is much more reliable than a blood pressure reading taken during a single visit. The magnitude of this response is similar to that reported in prior studies with beetroot juice, which correlated the blood pressure responses to the increases in plasma nitrite concentrations [43], and by others using a nitrate-rich vegetable diet [44]. Similar blood pressure effects have been observed in hypertensive subjects [45]. However, it should be noted that hypertensive individuals tend to exhibit a greater reduction in blood pressure from antihypertensive drugs than normotensive individuals; we therefore expected smaller blood pressure lowering effects in our study because most of our participants were normotensive. Interestingly, while nitrate treatment reduced blood pressure in overweight control women, no significant effects were found on either blood pressure or heart rate in women with prior RYGB bariatric surgery. Similarly, nitrate treatment improved vascular endothelial function in controls, but not in women with prior bariatric surgery. Our findings in obesity support prior results indicating that beetroot juice improved the vascular function assessed by flow-mediated dilation in older individuals [46] and hypertensive patients [45,47]. We feel that the lack of nitrate-mediated improvement in vascular function in participants with prior RYGB bariatric surgery we here report is an important addition to the existing body of literature on this subject.

Our clinical study did not aim to define precise mechanisms involved in the beneficial cardiovascular effects of nitrate treatment. However, earlier clinical studies have demonstrated that oral nitrate administration increases the circulating concentrations of nitrate, enhances its bioactivation to nitrite in the oral cavity, stimulates the formation of nitrite-derived nitrogen oxides in the stomach, and thereby increases the circulating concentration of RSNOs [[18], [19], [20]]. Our results showed similar increases in plasma nitrate concentrations in overweight controls and in the bariatric surgery group. The effects observed following nitrite or nitrate administration, both in experimental and in clinical studies, are typically attributed to enzymatic conversion of nitrite to NO by tissue-based nitrite reductases [[48], [49], [50], [51]]. If this was true, one would expect functional effects to correlate with circulating nitrite concentrations. However, plasma nitrite increased even more dramatically (7.5-fold) in the bariatric group compared to overweight controls (3.1-fold) despite much more remarkable cardiovascular effects in the latter. These findings strongly suggest that the cardiovascular effects of nitrate may not be explained by increased circulating nitrite concentrations, as previously assumed [16,52], but likely involve an interaction with endogenous compounds to form NO-containing species such as RSNOs. These assumptions are in line with earlier experimental results in rodents demonstrating a close link between nitrite (and nitrate) administration, circulating RSNO concentrations and associated pharmacological effects [53] as well as studies in humans reporting clear differences in the hemodynamic effects of nitrite between oral and intravenous administration even if given in amounts that lead to comparable circulating levels [54].

It is of utmost importance to note that nitrate administration tended to increase RXNO concentrations and almost doubled RSNO concentrations in the control group, whereas the concentrations of these nitroso species decreased in the bariatric surgery group. The increases in RSNO after nitrate treatment reported here is supported by recent studies with nitrate in healthy human volunteers [[18], [19], [20]]. However, the decreases in both RXNO and RSNO concentrations after nitrate therapy in participants with prior RYGB bariatric surgery strongly suggest that this surgery markedly alters the handling of swallowed nitrite in the gastrointestinal tract. Indeed, increased gastric pH after RYGB surgery [29] may severely impair oral nitrite-induced gastric RSNO formation and the associated cardiovascular responses, as previously shown in studies using inhibitors of gastric acid secretion [16,17,55]. This is because the acidic conditions of the stomach are critical for the disproportionation of HNO_2_ and subsequent RSNO formation. Therefore, our results are consistent with the notion that the RYGB surgery increases gastric pH, prevents RSNO formation, and disrupts the cardiovascular benefits typically induced by nitrate administration. While nitrate increased both nitrite and RSNO in overweight controls, the lack of increase in RSNO concentrations and cardiovascular effects in study participants with prior RYGB surgery is also in line with the idea that RSNOs are potent vasodilators whose effects are potentiated by nitrite [56]. Taken together, our results suggest that nitrate-induced increases in RSNO concentrations and nitrosation of cardiovascular targets [24] may be more relevant than the increases in circulating nitrite concentrations to produce cardiovascular effects. Supporting this suggestion, there is growing evidence that oral nitrite-induced RSNO formation promotes the nitrosation of pharmacological targets including calcium/calmodulin-dependent protein kinase II γ and protein Kinase C, which are involved in the regulation of vascular tone [26,27], and other proteins involved in regulating cardiac function [24,25]. It is important to note that the modest increases in circulating RSNO concentrations (5–10 nM) we observed in the control group cannot fully explain the blood pressure lowering effects of nitrate treatment. While we cannot exclude the possibility of synergistic effects of concomitant increases in both RSNO and nitrite [56], the blood pressure responses observed are more likely the result of chronic effects on vascular tone due to the nitrosation of pharmacological targets [26,27], as discussed above.

A previous study in healthy volunteers had shown that pretreatment with esomeprazole blunted the blood pressure responses to oral nitrite (maximum drop 15 min after nitrite) without affecting the increases in RXNO concentrations, suggesting that RSNO did not mediate the blood pressure responses to oral nitrite administration [54]. However, RXNO levels were measured 30 min after nitrite administration, and thus after rather than before maximum blood pressure effects (about 6 mmHg drop) were observed, making it difficult to infer causality and complicating the interpretation of results. Moreover, the responses to acute nitrite administration may well differ significantly from those of nitrate administration for two weeks.

An interesting finding of the present study was that nitrate administration caused similar increases in plasma nitrate concentrations in both groups of participants, whereas plasma nitrite concentrations increased by approximately 300 nM in controls and 1000 nM in the bariatric group. The observation that circulating nitrate increased similarly in both groups would suggest that RYGB surgery has no major effects on the secretion of nitrate into saliva and its absorption from the gastrointestinal tract into blood. Although our study does not allow to explain why such a striking difference in nitrite levels was observed between groups, this is likely attributable to a complex interplay of multiple factors. A lower conversion of nitrite to NO (and possibly other nitrogen oxides) in the stomach of participants in the bariatric group likely plays a major role, as suggested by lower RSNO concentrations in this group, and allowed increased amounts of nitrite entering the circulation. While nitrite and nitrate are both absorbed in the upper portion of the gastrointestinal tract [6], specific information on how RYGB surgery may affect nitrite/nitrate uptake is lacking. Adding additional complexity to the pharmacokinetics of nitrate and nitrite, there is evidence that nitrate is secreted into the gut lumen, where it is converted to nitrite by intestinal bacteria, thus increasing circulating nitrite concentrations [57]. RYGB surgery may impact this mechanism as it changes gut microbiome composition and other mechanisms potentially affecting nitrite and nitrate concentrations [28]. Those other changes associated with the RYGB surgery, including modifications in oral nitrate-reductase activity [58], may modify the responses to nitrate and remain to be explored. Alternatively, a reduced gastric nitrosative capacity may shift the equilibrium between formation and utilization of circulating nitroso species in blood. Taken together, these alterations caused by the RYGB surgery may underlie the decreased steady-state levels of RXNO/RSNO we found after nitrate treatment in the bariatric surgery group.

Recent studies have shown that nitrate administration is associated with antioxidant effects [[37], [38], [39], [40],^59^], which may be mediated by nitrite itself or other NO-related species [60]. While nitrate treatment decreased TBARS and increased TAC in controls, we found no effects in the bariatric surgery group, again indicating that RYGB surgery blunts the beneficial effects of nitrate. It is clear that increased oxidative stress plays a major role in cardiovascular diseases [61], and therefore the lack of antioxidant responses in the bariatric group shows that this therapeutic approach to obesity may prevent beneficial effects of dietary nitrate and perhaps even that of endogenous nitrate. It is relevant to note that baseline values for outcome measures in the two groups of the present study showed no significant differences, except for TBARS concentrations, which were significantly lower in the bariatric surgery group compared with controls (P < 0.05). While this difference has been reported as an effect of RYGB surgery before [62] it remains unclear whether it could affect the responses to nitrate treatment.

Our findings may have important implications for patients who underwent RYGB surgery to treat obesity. As alluded to above, dietary nitrate increases nitrite concentrations in saliva, which generate NO [12,13] and nitroso/nitrosyl species in the acidic environment of the stomach [63]. This in turn increases gastric mucosal blood flow and mucus thickness [64], and enhances the bactericidal action of gastric juice [63]. Patients who underwent RYGB surgery may therefore be at increased risk of complications associated with impaired gastric NO-related mechanisms beyond increased cardiovascular risk. It is not clear at this time whether nitrite or RSNO drive most of the nitrate-induced functional and biochemical effects, and it is possible that multiple NO metabolites may interact to exert their beneficial effects. However, the direct association between the improvement in endothelial function, daytime systolic blood pressure, and total antioxidant capacity with the increases in RSNO concentrations suggest a causal relationship between those responses and RSNO, although total antioxidant capacity also correlated positively with plasma nitrite concentrations. Mechanistic studies should be carried out to better define the role of NO-related species in the responses to nitrate in the future.

Our study is not without limitations. Firstly, despite RYGB having been used extensively in the past, it has now been largely replaced by sleeve gastrectomy [65]. Whether our findings may be fully applicable to patients that undergo other modalities of bariatric surgery, such as the sleeve method, is unknown at present and remains to be investigated. Secondly, we did not evaluate the metabolic profile of study participants, and it is highly probable that this surgery induces other relevant metabolic alterations which warrants further investigation. Thirdly, we did not measure gastric pH in the present study. However, previous studies unequivocally demonstrated that gastric pH increases significantly after RYGB surgery [29], and there is no reason to believe that this would not have been the case with our study participants in the bariatric group. Finally, we compared the concentrations of circulating NO species at baseline to those after 14 days (2.5 h after the last dose) of oral nitrate treatment, and therefore the changes in NO metabolite concentrations we observed may reflect both chronic and acute effects. Moreover, our study has not taken possible day to day variability into consideration.

In conclusion, our results show that RYGB surgery modifies the enterosalivary processing of nitrate in a way that severely compromises RSNO formation by disrupting gastric nitrosation processes, and nitrate-induced antioxidant and blood pressure-lowering responses, which contribute to improved endothelial function. Our findings provide evidence for a critical role of gastrointestinal system integrity in mediating the pharmacological responses to nitrate treatment and suggest that similar interferences may occur with dietary or even endogenously produced nitrate. Obese patients treated with RYGB surgery may benefit from supplementary pharmacological strategies to restore the biochemical pathways that govern nitrate/nitrite bioactivation to support health.

## CRediT authorship contribution statement

**Jéssica Maria Sanches-Lopes:** Writing – review & editing, Writing – original draft, Validation, Project administration, Methodology, Investigation, Formal analysis, Data curation, Conceptualization. **Alessandra Cássia-Barros:** Writing – review & editing, Writing – original draft, Validation, Methodology, Investigation, Formal analysis, Data curation, Conceptualization. **Sandra Oliveira Conde-Tella:** Writing – review & editing, Writing – original draft, Validation, Methodology, Investigation, Formal analysis, Data curation, Conceptualization. **Eduardo Barbosa Coelho:** Writing – review & editing, Writing – original draft, Validation, Methodology, Investigation, Formal analysis, Data curation, Conceptualization. **Rafael Kemp:** Writing – review & editing, Writing – original draft, Methodology, Investigation, Formal analysis, Data curation, Conceptualization. **Riccardo Lacchini:** Writing – review & editing, Writing – original draft, Investigation, Formal analysis, Data curation, Conceptualization. **Martin Feelisch:** Writing – review & editing, Writing – original draft, Validation, Methodology, Formal analysis, Conceptualization. **Wilson Salgado Júnior:** Writing – review & editing, Writing – original draft, Validation, Methodology, Investigation, Formal analysis, Data curation, Conceptualization. **Jose Eduardo Tanus-Santos:** Writing – review & editing, Writing – original draft, Validation, Supervision, Resources, Project administration, Funding acquisition, Formal analysis, Conceptualization.

## Declaration of competing interest

None.

## Data Availability

Data will be made available on request.

## References

[1] Moncada S., Higgs A. (1993). The L-arginine-nitric oxide pathway. N. Engl. J. Med..

[2] Lundberg J.O., Weitzberg E. (2022). Nitric oxide signaling in health and disease. Cell.

[3] Pinheiro L.C., Tanus-Santos J.E., Castro M.M. (2017). The potential of stimulating nitric oxide formation in the treatment of hypertension. Expert Opin. Ther. Targets.

[4] Farah C., Michel L.Y.M., Balligand J.L. (2018). Nitric oxide signalling in cardiovascular health and disease. Nat. Rev. Cardiol..

[5] Omar S.A., Webb A.J., Lundberg J.O., Weitzberg E. (2016). Therapeutic effects of inorganic nitrate and nitrite in cardiovascular and metabolic diseases. J. Intern. Med..

[6] Lundberg J.O., Gladwin M.T., Ahluwalia A., Benjamin N., Bryan N.S., Butler A., Cabrales P., Fago A., Feelisch M., Ford P.C., Freeman B.A., Frenneaux M., Friedman J., Kelm M., Kevil C.G., Kim-Shapiro D.B., Kozlov A.V., Lancaster J.R., Lefer D.J., McColl K., McCurry K., Patel R.P., Petersson J., Rassaf T., Reutov V.P., Richter-Addo G.B., Schechter A., Shiva S., Tsuchiya K., van Faassen E.E., Webb A.J., Zuckerbraun B.S., Zweier J.L., Weitzberg E. (2009). Nitrate and nitrite in biology, nutrition and therapeutics. Nat. Chem. Biol..

[7] DeMartino A.W., Kim-Shapiro D.B., Patel R.P., Gladwin M.T. (2019). Nitrite and nitrate chemical biology and signalling. Br. J. Pharmacol..

[8] Bryan N.S., Ivy J.L. (2015). Inorganic nitrite and nitrate: evidence to support consideration as dietary nutrients. Nutr. Res..

[9] Butler A.R., Feelisch M. (2008). Therapeutic uses of inorganic nitrite and nitrate: from the past to the future. Circulation.

[10] Weitzberg E., Lundberg J.O. (2013). Novel aspects of dietary nitrate and human health. Annu. Rev. Nutr..

[11] Hyde E.R., Andrade F., Vaksman Z., Parthasarathy K., Jiang H., Parthasarathy D.K., Torregrossa A.C., Tribble G., Kaplan H.B., Petrosino J.F., Bryan N.S. (2014). Metagenomic analysis of nitrate-reducing bacteria in the oral cavity: implications for nitric oxide homeostasis. PLoS One.

[12] Benjamin N., O'Driscoll F., Dougall H., Duncan C., Smith L., Golden M., McKenzie H. (1994). Stomach NO synthesis. Nature.

[13] Lundberg J.O., Weitzberg E., Lundberg J.M., Alving K. (1994). Intragastric nitric oxide production in humans: measurements in expelled air. Gut.

[14] Pereira C., Barbosa R.M., Laranjinha J. (2015). Dietary nitrite induces nitrosation of the gastric mucosa: the protective action of the mucus and the modulatory effect of red wine. J. Nutr. Biochem..

[15] Rocha B.S., Gago B., Barbosa R.M., Cavaleiro C., Laranjinha J. (2015). Ethyl nitrite is produced in the human stomach from dietary nitrate and ethanol, releasing nitric oxide at physiological pH: potential impact on gastric motility. Free Radic. Biol. Med..

[16] Pinheiro L.C., Amaral J.H., Ferreira G.C., Portella R.L., Ceron C.S., Montenegro M.F., Toledo J.C., Tanus-Santos J.E. (2015). Gastric S-nitrosothiol formation drives the antihypertensive effects of oral sodium nitrite and nitrate in a rat model of renovascular hypertension. Free Radic. Biol. Med..

[17] Sanches-Lopes J.M., Ferreira G.C., Pinheiro L.C., Kemp R., Tanus-Santos J.E. (2020). Consistent gastric pH-dependent effects of suppressors of gastric acid secretion on the antihypertensive responses to oral nitrite. Biochem. Pharmacol..

[18] Abu-Alghayth M., Vanhatalo A., Wylie L.J., McDonagh S.T., Thompson C., Kadach S., Kerr P., Smallwood M.J., Jones A.M., Winyard P.G. (2021). S-nitrosothiols, and other products of nitrate metabolism, are increased in multiple human blood compartments following ingestion of beetroot juice. Redox Biol..

[19] Wei C., Vanhatalo A., Black M.I., Blackwell J.R., Rajaram R., Kadach S., Jones A.M. (2024). Relationships between nitric oxide biomarkers and physiological outcomes following dietary nitrate supplementation. Nitric Oxide.

[20] Wei C., Vanhatalo A., Kadach S., Stoyanov Z., Abu-Alghayth M., Black M.I., Smallwood M.J., Rajaram R., Winyard P.G., Jones A.M. (2023). Reduction in blood pressure following acute dietary nitrate ingestion is correlated with increased red blood cell S-nitrosothiol concentrations. Nitric Oxide.

[21] Silva-Cunha M., Lacchini R., Tanus-Santos J.E. (2024). Facilitating nitrite-derived S-nitrosothiol formation in the upper gastrointestinal tract in the therapy of cardiovascular diseases. Antioxidants.

[22] Oliveira-Paula G.H., Pinheiro L.C., Tanus-Santos J.E. (2019). Mechanisms impairing blood pressure responses to nitrite and nitrate. Nitric Oxide.

[23] Broniowska K.A., Hogg N. (2012). The chemical biology of S-nitrosothiols. Antioxidants Redox Signal..

[24] Lima B., Forrester M.T., Hess D.T., Stamler J.S. (2010). S-nitrosylation in cardiovascular signaling. Circ. Res..

[25] Neto-Neves E.M., Pinheiro L.C., Nogueira R.C., Portella R.L., Batista R.I., Tanus-Santos J.E. (2019). Sodium nitrite improves hypertension-induced myocardial dysfunction by mechanisms involving cardiac S-nitrosylation. J. Mol. Cell. Cardiol..

[26] Pinheiro L.C., Oliveira-Paula G.H., Ferreira G.C., Dal-Cin de Paula T., Duarte D.A., Costa-Neto C.M., Tanus-Santos J.E. (2021). Oral nitrite treatment increases S-nitrosylation of vascular protein kinase C and attenuates the responses to angiotensin II. Redox Biol..

[27] Oliveira-Paula G.H., R I.M.B., Stransky S., Tella S.C., Ferreira G.C., Portella R.L., Pinheiro L.C., Damacena-Angelis C., Riascos-Bernal D.F., Sidoli S., Sibinga N., Tanus-Santos J.E. (2023). Orally administered sodium nitrite prevents the increased alpha-1 adrenergic vasoconstriction induced by hypertension and promotes the S-nitrosylation of calcium/calmodulin-dependent protein kinase II. Biochem. Pharmacol..

[28] Heymsfield S.B., Wadden T.A. (2017). Mechanisms, pathophysiology, and management of obesity. N. Engl. J. Med..

[29] Porat D., Vaynshtein J., Gibori R., Avramoff O., Shaked G., Dukhno O., Czeiger D., Sebbag G., Dahan A. (2021). Stomach pH before vs. after different bariatric surgery procedures: clinical implications for drug delivery. Eur. J. Pharm. Biopharm..

[30] Collares-Pelizaro R.V.A., Santos J.S., Nonino C.B., dos Reis Dias L.A., Gaitani C.M., Salgado W. (2017). Omeprazole absorption and fasting gastrinemia after roux-en-Y gastric bypass. Obes. Surg..

[31] Conchon Costa A.C., Medeiros J.I.M., Kang W., Yamamoto P.A., de Gaitani C.M., Vasconcelos M.E.D., Da Silva R.M., Kemp R., Sankarankutty A.K., Salgado W., Santos J.S., Schmidt S., De Moraes N.V. (2024). Redefining statin dosage post-gastric bypass: insights from a population pharmacokinetics-pharmacodynamics link approach. J. Clin. Pharmacol..

[32] Bjorne H., Govoni M., Tornberg D.C., Lundberg J.O., Weitzberg E. (2005). Intragastric nitric oxide is abolished in intubated patients and restored by nitrite. Crit. Care Med..

[33] Lima-Silva A.K., Rebelo M.A., Barros A.C., Conde-Tella S.O., Tanus-Santos J.E. (2024). The skeletal muscle, the heart, and the liver are the major organs of the accumulation of nitric oxide metabolites after oral nitrite treatment. Antioxidants.

[34] Flammer A.J., Anderson T., Celermajer D.S., Creager M.A., Deanfield J., Ganz P., Hamburg N.M., Luscher T.F., Shechter M., Taddei S., Vita J.A., Lerman A. (2012). The assessment of endothelial function: from research into clinical practice. Circulation.

[35] Feelisch M., Rassaf T., Mnaimneh S., Singh N., Bryan N.S., Jourd'Heuil D., Kelm M. (2002). Concomitant S-, N-, and heme-nitros(yl)ation in biological tissues and fluids: implications for the fate of NO in vivo. Faseb. J..

[36] Pinheiro L.C., Ferreira G.C., Damacena de Angelis C., Toledo J.C., Tanus-Santos J.E. (2020). A comprehensive time course study of tissue nitric oxide metabolites concentrations after oral nitrite administration. Free Radic. Biol. Med..

[37] Bahadoran Z., Carlstrom M., Ghasemi A., Mirmiran P., Azizi F., Hadaegh F. (2018). Total antioxidant capacity of the diet modulates the association between habitual nitrate intake and cardiovascular events: a longitudinal follow-up in Tehran Lipid and Glucose Study. Nutr. Metab..

[38] Carlstrom M., Montenegro M.F. (2019). Therapeutic value of stimulating the nitrate-nitrite-nitric oxide pathway to attenuate oxidative stress and restore nitric oxide bioavailability in cardiorenal disease. J. Intern. Med..

[39] Ferrer M.D., Capo X., Reynes C., Quetglas M., Salaberry E., Tonolo F., Suau R., Mari B., Tur J.A., Sureda A., Pons A. (2021). Dietary sodium nitrate activates antioxidant and mitochondrial dynamics genes after moderate intensity acute exercise in metabolic syndrome patients. J. Clin. Med..

[40] Yang T., Zhang X.M., Tarnawski L., Peleli M., Zhuge Z., Terrando N., Harris R.A., Olofsson P.S., Larsson E., Persson A.E.G., Lundberg J.O., Weitzberg E., Carlstrom M. (2017). Dietary nitrate attenuates renal ischemia-reperfusion injuries by modulation of immune responses and reduction of oxidative stress. Redox Biol..

[41] Ohkawa H., Ohishi N., Yagi K. (1979). Assay for lipid peroxides in animal tissues by thiobarbituric acid reaction. Anal. Biochem..

[42] Rice-Evans C.A. (2000). Measurement of total antioxidant activity as a marker of antioxidant status in vivo: procedures and limitations. Free Radic. Res..

[43] Webb A.J., Patel N., Loukogeorgakis S., Okorie M., Aboud Z., Misra S., Rashid R., Miall P., Deanfield J., Benjamin N., MacAllister R., Hobbs A.J., Ahluwalia A. (2008). Acute blood pressure lowering, vasoprotective, and antiplatelet properties of dietary nitrate via bioconversion to nitrite. Hypertension.

[44] van der Avoort C.M.T., Jonvik K.L., Nyakayiru J., van Loon L.J.C., Hopman M.T.E., Verdijk L.B. (2020). A nitrate-rich vegetable intervention elevates plasma nitrate and nitrite concentrations and reduces blood pressure in healthy young adults. J. Acad. Nutr. Diet..

[45] Kapil V., Khambata R.S., Robertson A., Caulfield M.J., Ahluwalia A. (2015). Dietary nitrate provides sustained blood pressure lowering in hypertensive patients: a randomized, phase 2, double-blind, placebo-controlled study. Hypertension.

[46] Jones T., Dunn E.L., Macdonald J.H., Kubis H.P., McMahon N., Sandoo A. (2019). The effects of beetroot juice on blood pressure, microvascular function and large-vessel endothelial function: a randomized, double-blind, placebo-controlled pilot study in healthy older adults. Nutrients.

[47] Broxterman R.M., La Salle D.T., Zhao J., Reese V.R., Richardson R.S., Trinity J.D. (2019). Influence of dietary inorganic nitrate on blood pressure and vascular function in hypertension: prospective implications for adjunctive treatment. J. Appl. Physiol..

[48] Zweier J.L., Li H., Samouilov A., Liu X. (2010). Mechanisms of nitrite reduction to nitric oxide in the heart and vessel wall. Nitric Oxide.

[49] Gilchrist M., Shore A.C., Benjamin N. (2011). Inorganic nitrate and nitrite and control of blood pressure. Cardiovasc. Res..

[50] Ghosh S.M., Kapil V., Fuentes-Calvo I., Bubb K.J., Pearl V., Milsom A.B., Khambata R., Maleki-Toyserkani S., Yousuf M., Benjamin N., Webb A.J., Caulfield M.J., Hobbs A.J., Ahluwalia A. (2013). Enhanced vasodilator activity of nitrite in hypertension: critical role for erythrocytic xanthine oxidoreductase and translational potential. Hypertension.

[51] Oliveira-Paula G.H., Pinheiro L.C., Guimaraes D.A., Tella S.O., Blanco A.L., Angelis C.D., Schechter A.N., Tanus-Santos J.E. (2016). Tempol improves xanthine oxidoreductase-mediated vascular responses to nitrite in experimental renovascular hypertension. Redox Biol..

[52] Pinheiro L.C., Ferreira G.C., Amaral J.H., Portella R.L., Tella S.O.C., Passos M.A., Tanus-Santos J.E. (2016). Oral nitrite circumvents antiseptic mouthwash-induced disruption of enterosalivary circuit of nitrate and promotes nitrosation and blood pressure lowering effect. Free Radic. Biol. Med..

[53] Bryan N.S., Fernandez B.O., Bauer S.M., Garcia-Saura M.F., Milsom A.B., Rassaf T., Maloney R.E., Bharti A., Rodriguez J., Feelisch M. (2005). Nitrite is a signaling molecule and regulator of gene expression in mammalian tissues. Nat. Chem. Biol..

[54] Montenegro M.F., Sundqvist M.L., Larsen F.J., Zhuge Z., Carlstrom M., Weitzberg E., Lundberg J.O. (2017). Blood pressure-lowering effect of orally ingested nitrite is abolished by a proton pump inhibitor. Hypertension.

[55] Pinheiro L.C., Montenegro M.F., Amaral J.H., Ferreira G.C., Oliveira A.M., Tanus-Santos J.E. (2012). Increase in gastric pH reduces hypotensive effect of oral sodium nitrite in rats. Free Radic. Biol. Med..

[56] Liu T., Zhang M., Terry M.H., Schroeder H., Wilson S.M., Power G.G., Li Q., Tipple T.E., Borchardt D., Blood A.B. (2018). Nitrite potentiates the vasodilatory signaling of S-nitrosothiols. Nitric Oxide.

[57] Eriksson K.E., Yang T., Carlstrom M., Weitzberg E. (2018). Organ uptake and release of inorganic nitrate and nitrite in the pig. Nitric Oxide.

[58] Ahmed K.A., Kim K., Ricart K., Van Der Pol W., Qi X., Bamman M.M., Behrens C., Fisher G., Boulton M.E., Morrow C., O'Neal P.V., Patel R.P. (2021). Potential role for age as a modulator of oral nitrate reductase activity. Nitric Oxide.

[59] Ashor A.W., Chowdhury S., Oggioni C., Qadir O., Brandt K., Ishaq A., Mathers J.C., Saretzki G., Siervo M. (2016). Inorganic nitrate supplementation in young and old obese adults does not affect acute glucose and insulin responses but lowers oxidative stress. J. Nutr..

[60] Amaral J.H., Rizzi E.S., Alves-Lopes R., Pinheiro L.C., Tostes R.C., Tanus-Santos J.E. (2019). Antioxidant and antihypertensive responses to oral nitrite involves activation of the Nrf 2 pathway. Free Radic. Biol. Med..

[61] Griendling K.K., Camargo L.L., Rios F.J., Alves-Lopes R., Montezano A.C., Touyz R.M. (2021). Oxidative stress and hypertension. Circ. Res..

[62] Venancio F.A., Almeida L.A., Zovico P.V., Barauna V.G., Miguel G.P.S., Pedrosa R.G., Haraguchi F.K. (2021). Roux-en-Y gastric bypass and sleeve gastrectomy differently affect oxidative damage markers and their correlations with body parameters. Obes. Surg..

[63] Bjorne H., Weitzberg E., Lundberg J.O. (2006). Intragastric generation of antimicrobial nitrogen oxides from saliva--physiological and therapeutic considerations. Free Radic. Biol. Med..

[64] Lundberg J.O., Weitzberg E., Gladwin M.T. (2008). The nitrate-nitrite-nitric oxide pathway in physiology and therapeutics. Nat. Rev. Drug Discov..

[65] Ozsoy Z., Demir E. (2018). Which bariatric procedure is the most popular in the world? A bibliometric comparison. Obes. Surg..

